# Effect of Hypoxia-Inducible Factor 1*α* on Early Healing in Extraction Sockets

**DOI:** 10.1155/2018/8210637

**Published:** 2018-05-08

**Authors:** Hyun-Chang Lim, Daniel S. Thoma, Mijeong Jeon, Je-Seon Song, Sang-Kyou Lee, Ui-Won Jung

**Affiliations:** ^1^Clinic for Fixed and Removable Prosthodontics and Dental Material Science, University of Zurich, Zurich, Switzerland; ^2^Department of Pediatric Dentistry, Oral Science Research Center, College of Dentistry, Yonsei University, Seoul, Republic of Korea; ^3^Department of Biotechnology, College of Life Science and Biotechnology, Yonsei University, Seoul, Republic of Korea; ^4^Department of Periodontology, Research Institute for Periodontal Regeneration, College of Dentistry, Yonsei University, Seoul, Republic of Korea

## Abstract

The aim of the present study was to investigate the effect of hypoxia-inducible factor 1*α* (HIF1A) on the early healing (4 weeks) of extraction sockets exhibiting partial loss of the labial bone. Two extraction sockets of the maxillary incisors from each of six dogs were assigned to two treatment modalities: deproteinized bovine bone mineral (i) with 10% collagen (DBBM-C) soaked with HIF1A and covered by a collagen membrane (CM) (HIF group) or (ii) treated with DBBM-C only and covered by a CM (control group). Microcomputed tomography revealed some degree of collapse of the labial contour. The totally augmented volume and new bone volume did not differ significantly between two groups (*P* > 0.05). The histological analysis revealed that the apical area of the socket was mostly filled with newly formed bone, while there was less newly formed bone in the coronal area and incomplete cortex formation. The histomorphometric analysis revealed that the area of newly formed bone was significantly larger in the HIF group than the control group (12.16 ± 3.04 versus 9.48 ± 2.01 mm^2^, *P* < 0.05), while there was no significant intergroup difference in the total augmented area. In conclusion, even though DBBM-C soaked with HIF1A enhanced histomorphometric bone formation, this intervention did not demonstrate superiority in preventing ridge shrinkage compared to DBBM-C alone. Clinical relevance of these findings should be further studied.

## 1. Introduction

The interest in counteracting ridge shrinkage has increased in recent years [1], which has led to detailed investigations of so-called alveolar ridge preservation (ARP) using a variety of protocols and biomaterials [2]. A gold standard has yet to be established, even though many preclinical and clinical studies have demonstrated that ARP reduces ridge shrinkage compared to a naturally healed socket [1].

Previous clinical studies regarding ARP have generally used a healing period after ARP of more than 3 months before implant placement [3]. Although such period was used to ensure maturation of newly formed hard tissue, ARP may delay the overall treatment time [4]. A systematic review also suggested that ARP procedures might not be able to accelerate or keep up with natural healing [3].

Another criticism of ARP is the possibility of further augmentation at the time of implant placement [1], which is mainly due to ARP not completely preventing ridge shrinkage. Moreover, most clinical studies have targeted sockets with minimal destruction, with even further augmentation sometimes being reported [5]. It is reasonable to suspect that further augmentation is more likely for damaged sockets.

Enhancers for bone formation may be required in practical applications to address the above-mentioned issues. Bone morphogenetic protein-2, platelet-derived growth factor, and enamel matrix derivative have previously been utilized [6–8], but their effects have been somewhat unclear.

The establishment of a vascular network precedes the formation of mineralized tissue. Insufficient vascularity will inevitably interrupt the nutritional and metabolic supply, leading to compromised healing [9]. Hypoxia-inducible factor 1*α* (HIF1A) is able to stimulate angiogenesis by activating genes encoding proangiogenic factors [10, 11] and enhance new bone formation and bone mineral density [12–14]. Such angiogenic-osteogenic coupling has been tested in bone fracture, osteoporosis, and distraction osteogenesis models [12, 13, 15–17], suggesting that HIF1A has potential in bone tissue engineering. However, to the best of the present authors' knowledge, HIF1A has yet to be investigated in the field of ARP.

Previously, Jeon et al. (2017) induced HIF1A overexpression using novel protein transduction domain (PTD; Hph-1-GAL4, ARVRRRGPRRR) and demonstrated that PTD-induced HIF1A increased angiogenesis [18]. PTD is composed of short amino acid sequences of less than 30 bp and can penetrate the plasma membrane [19, 20], and thus it has been considered effective for delivering proteins, DNA/RNA, drugs, and biological factors to target cells [21]. The osteogenic potential of HIF1A assisted by PTD could be useful for addressing the above-described long and delayed healing and probability of further augmentation.

The aim of the present study was to investigate the effect of HIF1A on healing of sockets exhibiting partial loss of the labial bone plate at the early stage in dogs.

## 2. Materials and Methods

### 2.1. Animals

Six male beagle dogs weighing 10–12 kg were used for the present study (Gukje, Pocheon, Korea). An individual cage under standard laboratory condition was allowed for each dog. Daily monitoring by a veterinarian was provided throughout the study. The protocol for the animal experiments was approved by the Institutional Animal Care and Use Committee of Yonsei Medical Center, Seoul, Korea (IACUC Approval No. 2013-0317-4).

### 2.2. Study Design

Bilateral maxillary incisors (teeth #102 and #202) were chosen as the recipient sites. After extracting the teeth, a bone defect was created on the labial socket wall (4 mm wide and 6 mm high). The extraction sockets were randomly assigned to the following two groups: (i) treatment with deproteinized bovine bone mineral with 10% collagen (DBBM-C; Bio-Oss® Collagen, Geistlich Pharma, Wolhusen, Switzerland) soaked with 0.2 ml of HIF1A (4 *μ*g of HIF1A DNA was mixed with 100 *μ*g of Hph-1-GAL4 at room temperature for 15 min, and 0.2-ml aliquots of the solutions were used) and covered by a collagen membrane (CM; Bio-Gide®, Geistlich Pharma) (HIF group) or (ii) treatment with DBBM-C soaked with saline and covered by a CM (control group).

### 2.3. Experimental Materials: HIF1A and Hph-1-G4D

HIF1A was generated and Hph-1-G4D (GAL4-DBD) was purified as described by [18]. In brief,* Homo sapiens* HIF1A (NCBI Reference Sequence: NM_001530.3) was amplified using the polymerase chain reaction (PCR). The PCR product was inserted into the pEGFPN1 plasmid vector (Invitrogen, Carlsbad, CA, USA) using restriction enzyme NheI (Takara Bio, Otsu, Japan) at 5′ termini and KpnI (Takara Bio) at 3′ termini of the PCR fragment. The DNA of G4D combined with Hph-1 was transformed with* Escherichia coli* BL-21 Star (DE3) pLysS (Invitrogen). The recombinant proteins were subsequently mixed with SP Sepharose Fast Flow (GE Healthcare, Milwaukee, WI, USA) and Hph-1-G4D protein was eluted. The eluted proteins were desalted using PD-10 Sephadox B-25 (Amersham Pharmacia Biotech, Piscataway, NJ, USA) with 10% glycerol phosphate-buffered saline (Sigma-Aldrich, St Louis, MO, USA).

### 2.4. Animal Surgery

General anesthesia was induced by a subcutaneous injection of atropine (Kwangmyung Pharmaceutical, Seoul, Korea) and an intravenous injection of xylazine (Rompun, Bayer Korea, Seoul, Korea) and Zoletil (Virbac, Carros, France). Tracheal intubation for enflurane inhalation (Gerolan, Choongwae Pharmaceutical, Seoul, Korea) was performed. Surgical sites were locally anesthetized using 2% lidocaine HCl (Huons, Seoul, Korea).

**Figure 1:**
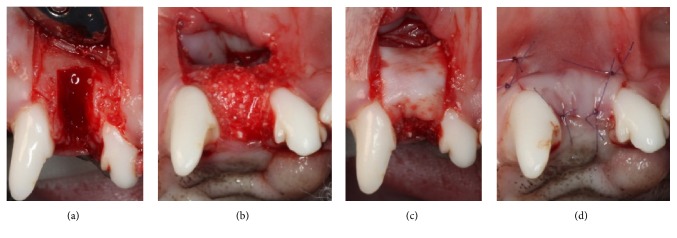
Clinical photographs of the surgical procedures. (a) Extraction and defect creation (4 mm wide and 6 mm high), (b) placement of either demineralized bovine bone mineral with 10% collagen (DBBM-C) soaked with hypoxia-inducible factor 1*α* (HIF1A) or DBBM-C only in the defect and the upper part of the socket, (c) coverage of the defect using a collagen membrane, and (d) primary flap closure.

Two vertical incisions were made at the mesial line angle of the mesial tooth and distal line angle of the distal tooth, and a sulcular incision was performed. Teeth #102 and #202 were carefully extracted, and defects were created on the labial aspect of the socket using a high-speed bur. Either DBBM-C soaked with HIF1A or DBBM-C soaked with saline (depending on the group allocation) was placed to fill the labial defect and the upper portion of the socket. DBBM-C was gently packed against the lingual wall and lightly squeezed between the lateral walls of the socket. Condensation into the apical direction was minimally performed. No labial overcorrection was performed. The defect and socket entrance were then covered by a CM. Primary flap closure was obtained through a periosteal releasing incision (Figure 1).

Antibiotic (20 mg/kg cefazoline, Yuhan, Seoul, Korea) was administered intramuscularly for 3 days postoperatively. The surgical wounds were disinfected daily using chlorhexidine (Bukwang, Seoul, Korea), and the animals were fed a soft diet throughout the healing period. After 4 weeks of healing, the dogs were euthanized by an overdose injection of pentobarbital sodium (90–120 mg/kg).

### 2.5. Microcomputed Tomography Analysis

The block sections of the experimental sites were harvested and immersed in 5% formic acid for 14 days. A microcomputed tomography (micro-CT) scan was performed (SkyScan 1072, SkyScan, Aartselaar, Belgium) at a resolution of 35 *μ*m (achieved using 100 kV and 100 *μ*A), and the acquired data were reconstructed with NRecon software (version 1.6.8.0, SkyScan, Kontich, Belgium).

#### 2.5.1. Volumetric Measurements

The binarization was conducted using the grayscale threshold values defined by ranging 115–225 for bone substitute and 69–115 for new bone. The following parameters were measured: total volume (TV) of the volume of interest, volume of newly formed bone (NV), and volume of residual bone substitute material (RV).

#### 2.5.2. Linear Measurements

The linear measurements were based on the assumption that the lingual plate of the socket would exhibit minimal resorption. A vertical reference line was drawn along the long axis in the center of each recipient socket, and perpendicular lines to this vertical reference were drawn at 1, 3, and 5 mm below the lingual crest. The horizontal width was determined at each of these levels, defined as HW_1_, HW_3_, and HW_5_.

### 2.6. Histological Processing and Histomorphometric Analysis

The resected specimens were then decalcified, trimmed, and embedded in paraffin. The blocks were sectioned serially in 5 *μ*m thickness perpendicular to the long axis of the socket. The central-most section was chosen for histological and histomorphometric analyses. Hematoxylin/eosin and Masson's trichrome staining were performed. The histological slides were scanned using digital slide scanner (Panoramic 250 Flash III, 3DHISTECH, Budapest, Hungary) and observed through CaseViewer (version 2.1, 3DHISTECH). The histomorphometric analysis was performed using CaseViewer (version 2.1, 3DHISTECH) and Photoshop CS6 (Adobe, CA, USA) by a single experienced investigator (H.C.L.) who was blinded to the group assignment.

**Figure 2:**
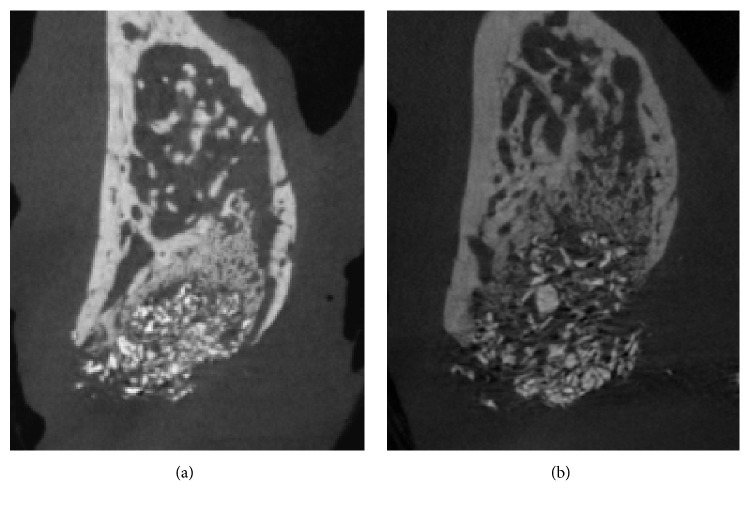
Representative microcomputed tomography (micro-CT) images: (a) control group and (b) HIF group.

The histomorphometric measurements were performed for both the entire augmented area and three rectangular regions of interest (ROIs) within the augmented area (each of size 2.0 mm^2^) set up by dividing the entire augmented area into three equal areas, defined as the coronal_1/3_, middle_1/3_, and apical_1/3_ areas. The following parameters were measured (Figure 2): (i) total augmented area including new bone, residual material, and nonmineralized tissue (TA), (ii) area of newly formed bone (NB), and (iii) area of residual bone substitute material (RM). The number of blood vessels (BV) was measured in each ROI.

### 2.7. Statistics

Statistical analyses were performed using a commercially available statistical package (SPSS 21.0, SPSS, Chicago, IL, USA). Data are presented as mean ± SD values. Shapiro-Wilk tests were used to check if the data conformed to a normal distribution, and then a paired *t*-test or the Wilcoxon signed-ranked test was applied. The cutoff for statistical significance was set at *P* < 0.05.

## 3. Results

### 3.1. Clinical Findings

Clinical healing was uneventful in all experimental animals. No adverse reaction such as pus discharge or swelling was observed.

### 3.2. Micro-CT Analysis

The labial contour at the coronal level of the socket generally shrunk in both the HIF and control groups. This tendency became pronounced from the margin of the defect to the labial crest. DBBM particles predominated in the upper half of the socket, with a small amount of newly formed bone between these particles. Some of the DBBM particles were displaced and scattered. Fewer DBBM particles were present in the lower half of the socket, with newly formed bone mostly occupying the space. Newly formed bone could still be differentiated from the socket wall due to its low radiopacity (Figure 2).

The horizontal width did not differ significantly between the HIF and control groups at any level (*P* > 0.05): HW_1_ was 5.79 ± 0.67 versus 5.47 ± 0.54 mm, HW_2_ was 6.71 ± 0.71 versus 6.70 ± 0.68 mm, and HW_3_ was 7.80 ± 0.50 versus 7.97 ± 0.58 mm.

**Figure 3:**
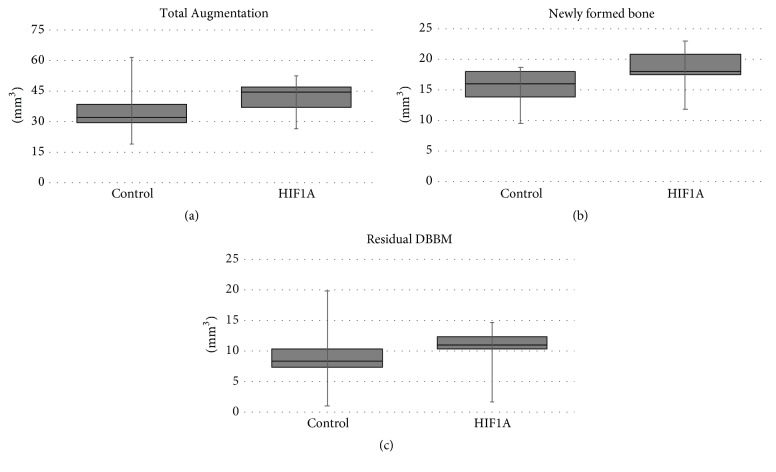
Parameters measured in the microcomputed tomography analysis: (a) total augmented volume (TV), (b) volume of newly formed bone (NV), and (c) volume of residual bone substitute material (RV). None of these parameters differed significantly between the two groups.

TV and NV were larger in the HIF group than the control group (41.58 ± 9.34 versus 35.48 ± 14.43 mm^3^ and 18.29 ± 3.94 versus 15.27 ± 3.47 mm^3^, resp.), but there was no significant intergroup difference (*P* > 0.05). RV also did not differ significantly between the HIF and control groups (10.18 ± 4.47 versus 9.17 ± 6.14 mm^3^, *P* > 0.05) (Figure 3, Table 1).

### 3.3. Histological Observations

**Figure 4:**
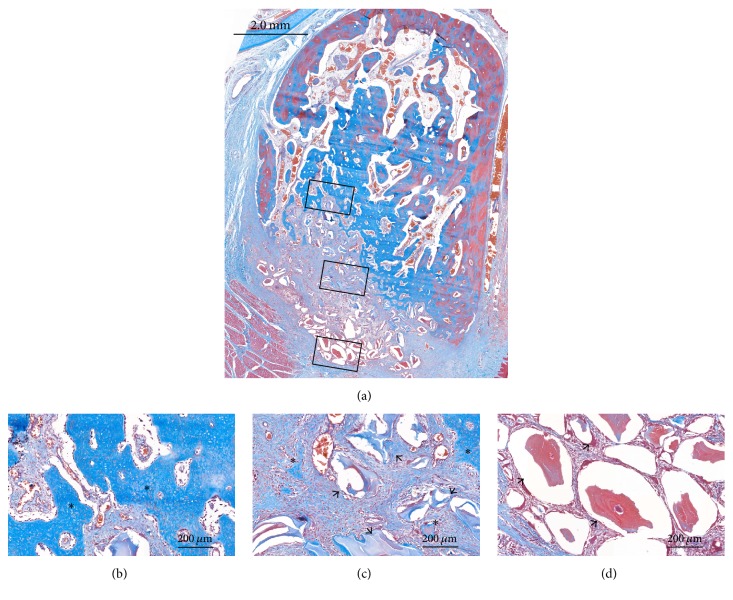
Histological views of the control group (Masson's trichrome stain). (a) Overall view of the alveolus. (b, c, d) High-magnification images of the boxed areas in the alveolus. ^*∗*^Newly formed bone; black arrow, residual bone substitute material.

**Figure 5:**
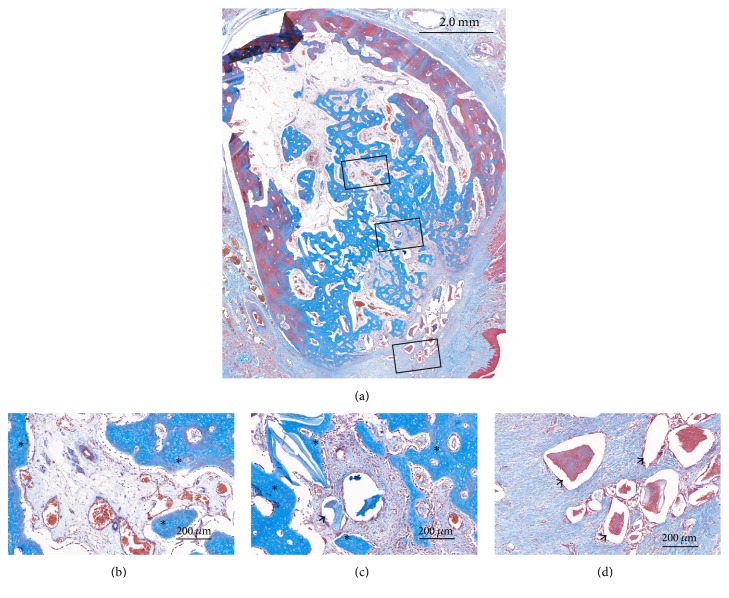
Histological views of the HIF group (Masson's trichrome stain). (a) Overall view of the alveolus. (b, c, d) High-magnification images of the boxed areas in the alveolus. ^*∗*^Newly formed bone; black arrow, residual bone substitute material.

At the coronal level of the ridge, the labial contour generally showed shrinkage in both groups, which started from the apical margin of the dehiscence defect. In contrast, the palatal bone plate remained almost unaffected. Most of the coronal area of the sockets was filled with DBBM. Some DBBM particles placed in the outermost area of the dehiscence defect were displaced and scattered in a few specimens (Figures 4 and 5).

The pattern of new bone formation was similar in the two groups, but the amount of newly formed bone appeared to be greater in the HIF group. New bone formation generally appeared to start from preexisting socket walls. There were finger-shaped projections of newly formed bone from the palatal, apical, and remaining labial socket walls. In the apical area, there were few DBBM particles, with it being filled by newly formed bone with osteocytes and reversal lines. Various amounts of DBBM particles were observed in the middle and coronal areas in both groups, but there appeared to be more particles in the control group. Newly formed bone and provisional matrix were observed on the DBBM particles in those areas (Figures 4 and 5).

Vascular structures of varying sizes were observed throughout the socket. Some blood vessels formed around the DBBM particles, but there were very few blood vessels around the particles in the outermost coronal part of the socket.

### 3.4. Histomorphometric Analysis

**Figure 6:**
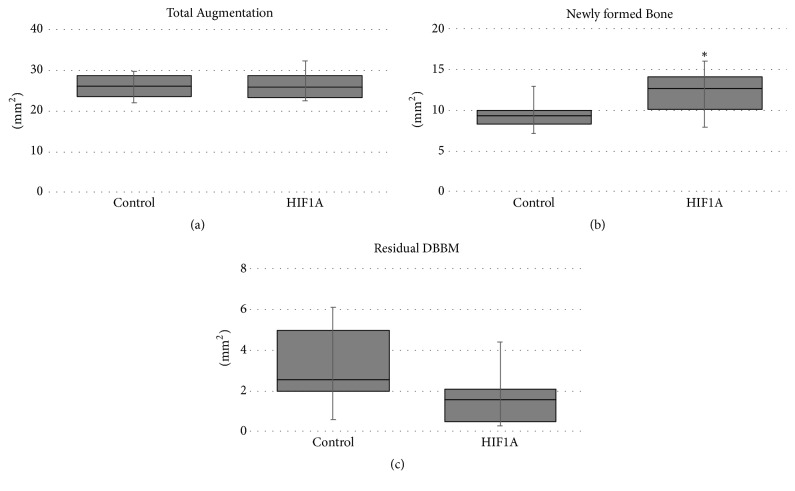
Parameters measured in the histomorphometric analysis: (a) total augmented area (TA), (b) area of newly formed bone (NB), and (c) area of residual bone substitute material (RM). ^*∗*^Significantly different compared to the control group.

TA did not differ significantly between the HIF and control groups (26.38 ± 3.88 versus 26.05 ± 3.21 mm^2^, *P* > 0.05). NB was significantly larger in the HIF group than the control group (12.16 ± 3.04 versus 9.48 ± 2.01 mm^2^, *P* = 0.042). RM was larger in the control group (3.22 ± 2.22 mm^2^) than the HIF group (1.69 ± 1.55 mm^2^), but there was no significant intergroup difference (Figure 6, Table 2).

In all ROIs (coronal_1/3_, middle_1/3_, and apical_1/3_ areas), NB and BV were larger in the HIF group than the control group, but there was no significant intergroup difference (*P* > 0.05). RM in all ROIs did not differ significantly between the HIF and the control group (*P* > 0.05) (Table 3).

## 4. Discussion

This study investigated whether or not HIF1A enhanced bone formation in extraction sockets exhibiting partial loss of the labial bone plate. Following 4 weeks of healing, it was demonstrated that (i) the histomorphometric amount of newly formed bone was significantly greater in the HIF group than the control group and (ii) the shrinkage of the labial contour was comparable in the two groups.

Many preclinical and clinical studies have investigated ARP [2, 22]. The results of previous studies appeared to be quite promising, but some disadvantages were also found, such as long healing periods after ARP and the possibility of further augmentation at the time of implant placement [4, 5]. Considering that angiogenesis always precedes osteogenesis, HIF1A might be one solution for overcoming these obstacles. Previous studies found that disruption of HIF1A in the osteoblasts led to thinner and less-vascularized bone [14] and that HIF1A injection enhanced gap healing following distraction osteogenesis [15]. These findings support angiogenic-osteogenic coupling by HIF1A. In line with those studies, the present study found greater new bone formation in the HIF group than the control group based on both histomorphometry (12.16 ± 3.04 versus 9.48 ± 2.01 mm^2^) and micro-CT (18.29 ± 3.94 versus 15.27 ± 3.47 mm^3^) analyses, although the difference was statistically significant only in the histomorphometric analysis.

However, irrespective of new bone formation, both groups showed shrinkage of the coronal area of the socket and with no significant difference in the resultant width. This result might be consistent with those from a previous comparison of ARP for different protocols (only DBBM, DBBM + CM, DBBM + rhBMP-2) with natural healing in sockets with the buccal bone removed [23]. Even though those authors found a significant intergroup difference in new bone formation, there was no difference in ridge shrinkage (approximately 20%) in the coronal area of ARP-received sockets. The addition of a barrier membrane and enhancers might improve the healing process from a histological point of view compared to simply filling the socket with bone substitute, but the maintenance of ridge dimension might not fulfill the expectations of clinicians.

In all of the present histological specimens, no cortex formation was observed in the labial area and the outermost part of the coronal area of the socket mainly consisted of DBBM particles. This healing pattern can be compared with the findings of De Santis et al. (2011) using the same recipient sites (canine maxillary incisors) [24]. They immediately placed external-type implants in extraction socket with a dehiscence defect on the labial aspect, performed guided bone regeneration with either autogenous bone or DBBM and a CM, and histologically examined the specimens after 8 and 16 weeks of healing. Those authors found that DBBM particles were located above the new alveolar crest of the defect and were not incorporated with bone matrix after 8 weeks, but the particles became in close contact with newly formed bone after 16 weeks. It can therefore be conjectured that DBBM particles located in the outermost part of the coronal area would be incorporated into the bone volume over time. A recent preclinical study also demonstrated that the above-mentioned immature tissue was capable of being modeled into bone tissue for implant placement during the early healing period after ARP [4]. However, it is disappointing that cortex formation still requires a sufficient healing time even when HIF1A is used.

It was expected that HIF1A would increase angiogenesis in the socket. BV was slightly higher in the HIF group than the control group, but there was no significant intergroup difference. This observation might be explained by several factors. First, during the surgery, DBBM-C was stabilized by squeezing into the dehiscence defect, but some scattering and displacement of the DBBM particles were observed in the histology and micro-CT analyses. This might have been due to uncontrolled pressure from the labial side, resorption of the collagen component in the DBBM-C, and no apical securement causing micromotion in the graft, since it was demonstrated that micromotion during the early healing period could favor fibrous tissue that lacks blood vessels [25]. Second, no delivery protocol for applying HIF1A has been verified in medium-sized and large animals. Jiang et al. (2016) locally injected two different doses of HIF1A (10 and 20 *μ*g) and saline daily into a distraction osteogenesis model in rabbit and found that the 20 *μ*g dose led to the highest mineralization [15]. The present study is the first to utilize DBBM-C for carrying HIF1A and Hph-1-G4D, and so further investigations are required.

One of the particularly interesting findings in the present study is related to the apical healing in both groups. During the surgery, DBBM-C was placed mainly in the labial defect area and the upper portion of the socket, and so the apical area received only a small amount of DBBM-C. After 4 weeks, there was abundant bone formation in the apical area, in contrast to the middle and coronal areas where most of the DMMB-C had been placed. This is in line with previous studies showing a complete filling of woven bone in the healing of nongrafted sockets at 4 weeks after tooth extraction [26, 27] and less woven bone formation in the socket filled with bone substitute material [28]. Clinically, these observations may question the necessity of apical filling. It has been clearly demonstrated that the most-susceptible area for ridge resorption following extraction is confined to the coronal area of the socket [29]. The area below the middle of the socket could remain stable without ARP, and so focused filling with a bone substitute material into the upper part of the socket may be feasible option for ARP.

The labial bone plates of the anterior teeth are prone to defects resulting from periodontal disease and trauma due to its natural thinness. In the present study, we therefore selected anterior teeth and tried to simulate sockets with defects by creating a dehiscence-type defect on the labial wall. Also, considering that immediate or early implant placement might be more straightforward than ARP for a socket with intact walls, the current model may be more relevant to many clinical situations. However, it should be noted that the present study used an acute type of defect, because the healing capacity differs between sockets with chronic pathologies and intact sockets [30].

The present study used both histomorphometric and micro-CT data to evaluate the effects of HIF1A. Although the general trends of newly formed bone were similar in these two types of analysis, statistically significant results were only detected in histomorphometry. The trend was somewhat opposite for residual bone substitute material. This kind of discrepancy was also previously noted [31]. Micro-CT analyses sometimes appear to be less sensitive because they require different ranges of grayscale values to be chosen for various tissues, and when a bone substitute is mixed with living bone tissue, the grayscale range for the bone substitute could overlap that for bone tissue. Care is therefore needed when interpreting the results from both types of analysis.

## 5. Conclusion

In conclusion, new bone formation was enhanced histomorphometrically when using DBBM-C with HIF1A compared to DBBM-C alone in sockets exhibiting partial loss of the labial bone plate. However, clinical relevance of this difference should be carefully interpreted due to small amount of difference and short healing period. Moreover, DBBM-C with HIF1A was not superior to DBBM-C alone in preventing ridge shrinkage in the coronal part of the socket.

## Figures and Tables

**Table 1:** Microcomputed tomographic data.

	Control	HIF1A	*P* value
TV (mm^3^)	35.48 ± 14.43	41.58 ± 9.34	0.300
NV (mm^3^)	15.27 ± 3.47	18.29 ± 3.94	0.120
RV (mm^3^)	9.17 ± 6.14	10.18 ± 4.47	0.685

Data are expressed as mean ± SD; TV, total volume of the volume of interest; NV, the volume of newly formed bone; RV, the volume of residual bone substitute material.

**Table 2:** Histomorphometric data of the entire socket.

	Control	HIF1A	*P* value
TA (mm^2^)	26.05 ± 3.21	26.38 ± 3.88	0.886
NB (mm^2^)	9.48 ± 2.01	12.16 ± 3.04	0.042
RM (mm^2^)	3.22 ± 2.22	1.69 ± 1.55	0.080

Data are expressed as mean ± SD; TA, total augmented area including new bone, residual material, and nonmineralized tissue; NB, the area of newly formed bone; RM, the area of residual bone substitute material.

**Table 3:** Histomorphometric data of the three regions of interest (ROIs) within the socket.

	Control	HIF1A	*P* value
NB (mm^2^)			
Coronal_1/3_	0.46 ± 0.50	0.67 ± 0.39	0.463
Middle_1/3_	0.65 ± 0.49	1.01 ± 0.19	0.204
Apical_1/3_	1.01 ± 0.42	1.08 ± 0.27	0.767
RM (mm^2^)			
Coronal_1/3_	0.66 ± 0.42	0.26 ± 0.31	0.141
Middle_1/3_	0.33 ± 0.30	0.07 ± 0.11	0.080
Apical_1/3_	0.00 ± 0.01	0.06 ± 0.14	0.655
BV (*n*)			
Coronal_1/3_	26.17 ± 11.70	30.33 ± 10.11	0.320
Middle_1/3_	28.67 ± 8.31	36.50 ± 10.09	0.108
Apical_1/3_	29.00 ± 13.91	31.50 ± 16.17	0.207

Data are expressed as mean ± SD. The dimension of each ROI was 2.0 mm^2^. NB, the area of newly formed bone; RM, the area of residual bone substitute material; BV, the number of blood vessels.
